# Immunohistochemichal Assessment of the CrkII Proto-oncogene Expression in Common Malignant Salivary Gland Tumors and Pleomorphic Adenoma

**DOI:** 10.15171/joddd.2015.006

**Published:** 2015-03-04

**Authors:** Mitra Askari, Masoud Darabi, Esa Jahanzad, Zahra Mostakhdemian Hosseini, Marjan Musavi Chavoshi, Maryam Darabi

**Affiliations:** ^1^Assistant Professor, Department of Oral & Maxillofacial Pathology, Faculty of Dentistry, Tehran University of Medical Sciences, International Campus, Tehran, Iran; ^2^Assistant Professor, Department of Biochemistry and Clinical Laboratories, School of Medicine, Tabriz University of Medical Sciences, Tabriz, Iran; ^3^Associate Professor, Department of Pathology, Faculty of Medicine, Tehran University of Medical Sciences, Tehran, Iran; ^4^Assistant Professor, Iran Tumor Bank, Cancer Institute, Imam Hospital, Tehran University of Medical Sciences, Tehran, Iran; ^5^Student of Dentistry, Faculty of Dentistry, Tehran University of Medical Sciences, International Campus, Tehran, Iran; ^6^Assistant Professor, Dental and Periodontal Research Center, Faculty of Dentistry, Tabriz University of Medical Sciences, Tabriz, Iran

**Keywords:** CrkII, immunohistochemistry, salivary gland carcinoma

## Abstract

***Background and aims.*** Various morphologies are seen in different salivary gland tumorsor within an individual tumor, and the lesions show divers biological behaviors. Experimental results support the hypothesis that increased CrkII proto-oncogene is associated with cytokine-induced tumor initiation and progression by altering cell motility signaling pathway. The aim of this study was to assess the CrkII expression in common malignant salivary gland tumors and pleomorphic ade-noma.

***Materials and methods.*** Immunohistochemical analysis of CrkII expression was performed on paraffin blocks of 64 car-cinomas of salivary glands, 10 pleomorphic adenomas, and 10 normal salivary glands. Biopsies were subjected to immu-nostaining with EnVision detection system using monoclonal anti-CrkII. Evaluation of immunoreactivity of CrkII was based on the immunoreaction intensity and percentage of stained tumor cells which were scored semi-quantitatively on a scale with four grades 0 to 3. Kruskal-wallis test and additional Mann-Whitney statistical test were used for analysis of CrkII expression levels.

***Results.*** Increased expression of CrkII was seen (P=0.005) in malignant tumors including: mucoepidermoid carcinoma, adenoid cystic carcinoma, and carcinoma ex pleomorphic adenoma, but CrkII expression in acinic cell carcinoma was weak. CrkII expression in pleomorphic adenoma was weak or negative. A weak staining was sparsely seen in normal acinar serous cell.

***Conclusion.*** Increased expression of CrkII and its higher intensity of staining in tumors with more aggressive biologic behavior in carcinomas of salivary gland is consistent with a role for this proto-oncogene in salivary gland tumorigenesis and cancer progression.

## Introduction

Salivary gland carcinomas constitute about 5% of head and neck cancers.^1^ Various morphologies are seen in different tumor types or even within an individual tumors, and these lesions show divers biological behaviors.^2^ Therefore, the diagnosis and management of such tumors becomes difficult by mere evaluation of prognostic factors like gender, age, location, size, TNM-stage, and positive surgical margins as well as the histologic factors(tumor type, grade).^1-4^ Immunohistochemistry assessment is currently recommended as an additional prognostic and diagnostic factor besides the evaluation of other clinico-histopathological factors in some tumors. Evaluating the expression status of some tumor sup-pressor genes and proto-oncogenes as the markers of prognosis, differential diagnosis, and selecting therapies are currently being studied widely in sali-vary gland carcinomas. For example, increased Ki67 as a proliferative marker has shown an additional value in prediction of survival in various types of these tumors.^5-7^

CT10 regulator kinase (Crk) was first discovered as V-Crk oncogene product in avian retroviruses.^8^ It has been shown that Crk could transform mammalian cells in several characteristics like focal adhesion, lamelliopadia formation, cell motility, and epithelial-mesenchymal-like transition.^9,10^ It is involved in signaling cascades induced by various stimuli including integrin molecules and cytokines.^11^ Crk family can be subdivided into two classes, CrkI and CrkII, based on sequence homology.^12^CrkII is a member of the adapter proteins which contain Src homology-2 (SH2) and Src homology-3 (SH3) domains.^13^ CrkII plays a key role in intracytoplasmic signaling by tyrosine phosphorylation of cellular proteins.

In addition to dysregulated Crk expression in some malignant neoplasms like glioblastoma and lung cancer,^14,15^ increased mRNA expression of CrkII genes was seen in more advanced stages of lung tumors with poor survival rates.^16^ CrkII has several important functions that involve regulation of the actin cytoskeleton, phagocytic entry of apoptotic cells, and pathogens into the host cells, as well as cell cycle, apoptosis, andmetabolism.^12^

In the last decade, the role of CrkII and CrkI in human cancers has extended the reliability of this protein as a tumor marker; however, no studies have been carried out to elucidate the expression pattern of CrkII in salivary gland tumors. According to the well-recognized role of CrkII in breast cancer and its strong correlations with breast cancer staging and also the similarity of secretory carcinoma of breast and salivary gland acinic cell carcinoma,^17^ we decided to find the expression pattern of Crk II in different salivary gland tumors. Therefore, this study was designed to investigate the expression level of CrkII in common malignant salivary glands tumors with different biologic behaviors and comparison with the most common benign salivary gland tumor (pleomorphic adenoma) and normal salivary gland tissues.

## Materials and Methods

Tumor biopsies were obtained from 64 patients (31 female, 33 male) who were operated between 2001 and 2006 at the University Hospital. The samples include 10 pleomorphic adenomas (PA), 11 acinic cell carcinomas (AcCC), 18 mucoepidermoid carcinomas (MEC), 12 carcinoma ex-pleomorphic adenomas (CaexPA) and 23adenoid cystic carcinomas (AdCC) paraffin blocks from patients with salivary glands neoplasm. Ten normal tissue samples of salivary gland from radical neck dissection surgery were also examined as normal controls.

### Immunohistochemistry

Four micrometers paraffin embedded tissue sections were mounted on glass slides. After the deparaffinization step in xylene, slides were immersed in target retrieval buffer solution (Dako, Glostrup, Denmark). In the next step for antigen retrieval, slides were autoclaved to 121°C for 5 min. Endogenous peroxidase were blocked by incubation in methanol containing 0.3% H_2_O_2_ for 30 min. Immunohistochemical staining was performed using EnVision System (EnVision+, Dako, Carpentaria, CA). The slides were incubated overnight at 4°C with primary antibody directly against CrkII (H-53, Santa Cruz Biotech, Inc., CA). The slides were immersed in diaminobenzidine (DAB) for signal visualization. Negative control was prepared by replacing the primary antibody with phosphate buffered saline (PBS).

Each slide was semi-quantitatively assigned a score of 0, 1, 2 or 3. Immunoreactivity of CrkII was based on intensity staining pattern of nuclei and cytoplasm with anti CrkII and percentage of stained tumor cells which were analyzed by consensus of two pathologists, as follows: 0, no staining; 1, weak to moderate in <25% of tumor cells; 2, moderate to severe in 25-50% of tumor cells; and 3, strong staining in >50% tumor cells. Scores ≥2 was defined as over expression.^18,19^

### Statistical Methods

Kruskal-Wallis test was used for analysis of CrkII expression levels. To compare CrkII expression levels among different tumor groups, additional Mann-Whitney U statistical test was used. A Pvalue of less than 0.05 was considered to be statistically significant.

## Results

The frequency distribution of cancer types showed no statistically significant difference in the studied population according to the gender or age group (Table 1).

**Table 1:** The frequency distribution of cancer types according to the age group and gender in 64 studied patients

Variable	Tumor type					Total	P value
	PA	AcCC	MEC	CaexPA	AdCC		
Gender, n							
Male	4	10	9	7	7	4	0.67
Female	6	13	9	5	4	6	
Age group, n							
>60 y	7	20	17	6	2	7	0.48
≤60 y	3	3	1	6	9	3	

### CrkII Expression in the Normal Tissue of Salivary Gland

**Figure 1.**
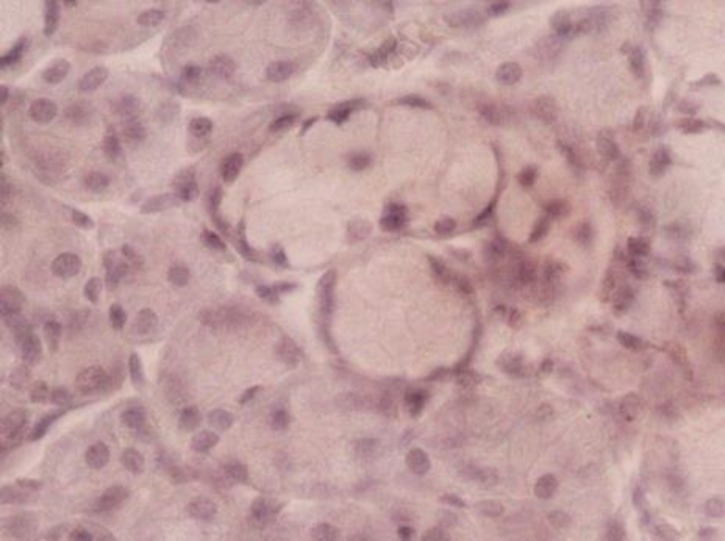
Representative immunohistochemical staining of CRKII in normal salivary gland

Staining was not seen in normal salivary glands (Table 2). A weak staining was detected in some cases of acinar serous cells, and ductal epithelial cells showed no staining (Figure 1).

**Table 2:** Cross-tabulation of cytoplasmic and membrane CrkII staining in salivary gland biopsies

Lesion, (n)	Staining score				Total
	Non, 0	Weak, 1	Moderate, 2	Strong, 3	
Normal salivary gland	6	4	0	0	10
Salivary gland carcinoma					
Pleomorphic adenoma (PA)	5	4	1	0	10
Acinic cell carcinomas (AcCC)	2	6	3	0	11
Mucoepidermoid carcinomas (MEC)	1	1	4	12	18
Carcinoma ex pleomorphic adenomas (CaexPA)	0	0	4	8	12
Adenoid cystic carcinomas (AdCC)	2	4	4	13	23
Total	10	15	16	33	74

### CrkII Expression in PA

**Figure 2.**
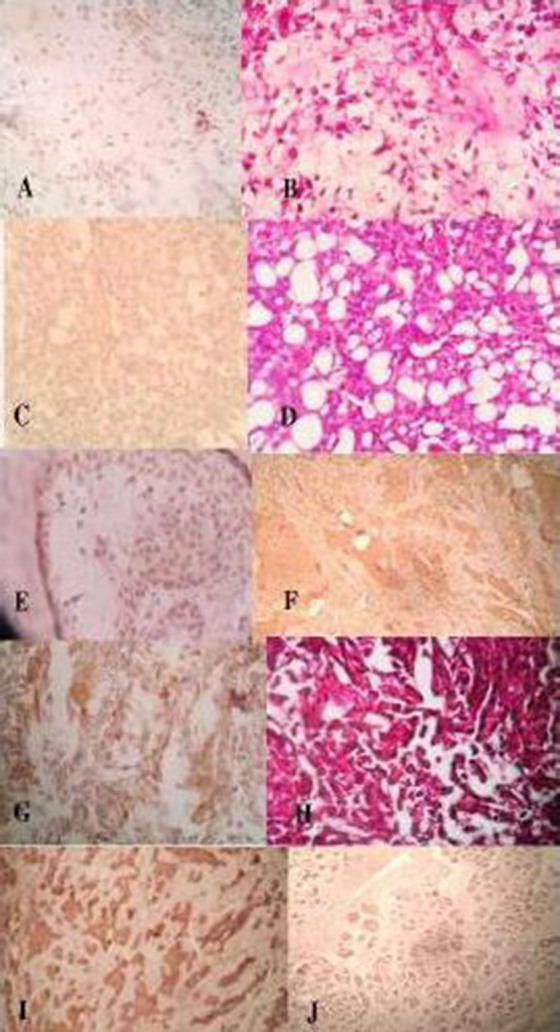
Representative immunohistochemical staining of CrkII (magnification, ×10). H-E staining (B, D, H) and immunohistochemical staining of CrkII (A, C, E, F, G, J and I). Weak staining in pleomorphic adenoma (A, B), CrkII expression in acinic cell carcinoma (C, D), strong cytoplasmic and membrane epithelial staining in mucoepidermoid carcinoma (E, F), strong cytoplasmic and membrane staining in ex pleomorphic adenoma (G, H), strong cytoplasmic and membrane staining (staining score 3) in adenoid cystic carcinoma (I, J).

Only 5 of the 10 cases of PA cases showed a weak staining and CrkII expression was totally absent in other PA specimens (Figure2A, B).

### CrkII Expression in Salivary Glands Carcinomas

Expression of CrkII Significantly increased in MEC, CaexPA and AdCC in comparison with normal salivary gland tissue and PA (P=0.005; Figure 2 G, h, E, F, I, J);however, in the AcCC, CrkII expression level was not significant in comparison with PA and normal salivary glands tumor (Figure 2 C, D). No significant difference was observed between the three other lesions (MEC, CaexPA, AdCC).

In MECs, cytoplasmic and membrane staining was negative in the mucousal tumor cells. Intensity of staining in tumors with higher epithelial cells were more than the tumors with higher mucosal cells (P=0.001).

Additionally, the intensity of staining was pronounced in epithelial cells with more pleomorphic and atypical changes, and in invasive areas (front).

In all cases, cytoplasmic staining was seen; however, nuclear staining was not seen. In AdCC, no differences were seen in cribriform, tubular, or solid histologic types.

Staining was more in ExPA cells with malignant changes and in areas that were more associated with the invasion to surrounding tissue.

## Discussion

Given the potential role of CrkII in tumor initiation and progression, we tested the CrkII expression in malignant salivary gland tumors and PA. The results of our study showed significantly increased expression of CrkII in MEC, CaexPA and AdCC in comparison with normal salivary gland tissue, PA and AcCC. These findings support the hypothesis that this proto-oncogene may alter the signaling pathway of tyrosine phosphorylation and perhaps also some cellular processes like cell growth and cell motility which play a key role in cancer metastasis and migration.

To the best of our knowledge, no research has addressed the role and mechanism of CrkII in salivary gland tumours to date; however, Rodrigues et al^20^have shown that CrkI/II knock down in breast cancer, a condition relatively similar to salivary glands carcinoma,^17^ could decrease cell spreading on extracellular matrix. The mechanism mainly related to the ability of CrkII in cell spreading is reducing actin fiber stress and formation of focal adhesion by the ability of Crk II SH2 domain to bind specific related tyrosine kinases. The study by Pasquale et al^21^shows that suppression of migration of the human malignant breast cancer by EphrinB2 requires phosphorylation of CrkII on Tyr221 by Abl which could possibly disassemble Crk II mediated p130cas/ Crk/DOCK180 complex.

CrkII does not have any intrinsic tyrosine kinase activity. However, it has been shown that increased expression level of adaptor protein CrkII could alter the cell signaling pathway by the two Src homology domains SH2 and SH3.^10-13^ These domains are responsible for assembly of protein-protein complexes.^22^ Interactions of the SH2 domain of CrkII with specific phosphorylated tyrosine residue motif like YXXXP play a major role in some cellular processes like cell growth and development, cell motility, focal adhesion and migration.^23,24^ Studies have shown increased mRNA expression and protein level of CrkII in various types of cancers like glyioblastoma and lung cancer.^15^ Analysis of different specimens of lung adenocarcinomas and breast tumors have also shown a significant increase of CrkII in stage III compared to stage I.^19^ We also noted that CrkII expression levels in low grade carcinoma AcCC as well as in benign neoplasm PA of salivary gland was less than those in invasive and metastatic salivary gland tumors. These findings are mostly consistent with the results of previous studies by Yamada et al^18,19^ which show overexpression of CrkII is strongly correlated with higher grades in T classification of cancers.

Cell movement is a complex phenomenon which is dependent on cytoskeleton actin remodeling. It has been shown that recognizing specific phosphorylated tyrosine residues on p130Cas and paxillin by SH2 domain of Crk and DOCK180 by SH3 domain of Crk will activate cell signaling pathway, which triggers cytoskeketal movement and cancer cell invasion and migration.^25,26^ Thus, increased expression of CrkII in CaexPA, MEC and AdCC are directly associated with enhanced invasive and metastatic properties of these tumors.

Expression analysis in kidney has shown that CrkII plays a key role in the acquisition of a mesenchymal phenotype by epithelial cells. Down regulation or mutation of E-cadherin or β-catenin will lead to acquisition of a motile mesenchymal phenotype in the MDCK kidney epithelial cells.^27,28^ These data are in agreement with our observation of more CrkII expression in cells with higher malignant changes and in invasive areas inMEC and ExPA groups.

Regarding to a great similarity between cell of salivary and mammary gland tumors and the crucial role of CRKII in morphogenesis, migration, invasion and motility of cells in many of malignancy like breast cancer.^29^

We investigated the expression status of CrkII, and its possible relationship with invasion and metastasis in salivary gland tumors. This study reports CrkII protein expression in carcinomas of salivary glands and provides evidence that the CrkII proto-oncogene may plays an important function in aggressiveness of salivary gland cancers. Recent studies described two roles for CrkII, as a marker in tumor cells.^30^ First, CrkII may be used as a potential tool for prognosis of cancers. Second, it may serve as a therapeutic target in some cancers like glioblastoma and breast cancer using RNAitechnology.^16^ It has been found that siRNA-mediated gene silencing of Crk significantly reduced migration and invasive behavior of tumor cells in both glioblastoma^16,30^ and breast cancer.^31^ However, there seems to be no study on the status of CrkII expression in malignant and benign tumors of salivary glands.

In conclusion, increased expression of CrkII and its higher expression in the cells with higher malignant changes and in invasive areas (front) at carcinomas of salivary gland with more aggressive biologic behavior may be consistent with a role for this proto-oncogene in salivary gland tumorigenesis and cancer progression. Further studies are required to establish the role of CrkII suppression as a possible novel therapeutic approach for salivary gland tumor metastasis. Also, investigating its relation with clinicohistopathologic factors influencing prognosis and treatment is recommended.

## Acknowledgement

This study was supported by a grant (388149) from Tehran University of Medical Sciences. A part of the study was performed as a DMD thesis. The authors acknowledge the Dental and Periodontal Research Center at Tabriz University of Medical Sciences.
